# Sex-specific offspring discrimination reflects respective risks and costs of misdirected care in a poison frog

**DOI:** 10.1016/j.anbehav.2016.02.008

**Published:** 2016-04

**Authors:** Eva Ringler, Andrius Pašukonis, Max Ringler, Ludwig Huber

**Affiliations:** a Messerli Research Institute, University of Veterinary Medicine Vienna, Medical University of Vienna, and University of Vienna, Vienna, Austria; b Department of Integrative Zoology, University of Vienna, Vienna, Austria; c Department of Cognitive Biology, University of Vienna, Vienna, Austria

**Keywords:** amphibians, offspring discrimination, parental care, sex differences, tadpole transport

## Abstract

The ability to differentiate between one's own and foreign offspring ensures the exclusive allocation of costly parental care to only related progeny. The selective pressure to evolve offspring discrimination strategies is largely shaped by the likelihood and costs of offspring confusion. We hypothesize that males and females with different reproductive and spatial behaviours face different risks of confusing their own with others' offspring, and this should favour differential offspring discrimination strategies in the two sexes. In the brilliant-thighed poison frog, *Allobates femoralis*, males and females are highly polygamous, terrestrial clutches are laid in male territories and females abandon the clutch after oviposition. We investigated whether males and females differentiate between their own offspring and unrelated young, whether they use direct or indirect cues and whether the concurrent presence of their own clutch is essential to elicit parental behaviours. Males transported tadpoles regardless of location or parentage, but to a lesser extent in the absence of their own clutch. Females discriminated between clutches based on exact location and transported tadpoles only in the presence of their own clutch. This sex-specific selectivity of males and females during parental care reflects the differences in their respective costs of offspring confusion, resulting from differences in their spatial and reproductive behaviours.

In species with parental care, the ability to recognize and discriminate between one's own offspring and unrelated young can have considerable fitness consequences for both the caregiving parent and its progeny (Beecher, 1991; Sherman, Reeve, & Pfennig, 1997). As parental behaviours are often very costly, parents in noncooperatively breeding species should ensure that care is directed exclusively to their own progeny (Duckworth, Badyaev, & Parlow, 2003; Queller, 1997; Trivers, 1972; but see also Larsson, Tegelström, & Forslund, 1995). Thus in several species males adjust the intensity of care according to the level of perceived paternity (bluegill sunfish, *Lepomis macrochirus*: Neff, 2003; eastern bluebirds, *Sialia sialis*: MacDougall-Shackleton & Robertson, 1998; pumpkinseed sunfish, *Lepomis gibbosus*: Rios-Cardenas & Webster, 2005; blue-footed boobies, *Sula nebouxii*: Osorio-Beristain & Drummond, 2001; but see also Kempenaers, Lanctot, & Robertson, 1998).

Substantial fitness benefits of accurate offspring discrimination abilities can be expected particularly when the risk of misdirected care is high (i.e. the likelihood of mistaking unrelated for one's own offspring, Westneat & Sherman, 1993). This is the case, for example, when offspring are highly mobile, when foreign progeny are in close spatial proximity, under polygamy or when cuckoldry is common. Several mechanisms have been proposed to explain how parents may differentiate between their own offspring and unrelated young: recognition alleles, phenotype matching, assortative learning or spatial recognition (Komdeur & Hatchwell, 1999; Sherman et al., 1997). Discrimination mechanisms are also classified regarding the use of direct or indirect cues: direct recognition refers to parents recognizing specific phenotypic characteristics of their young (chemical: Head, Doughty, Blomberg, & Keogh, 2008; Neff, 2003; Neff & Sherman, 2005; acoustic: Knörnschild & Von Helversen, 2008; visual: Lahti & Lahti, 2002; Underwood & Sealy, 2006); indirect recognition occurs if parents use contextual cues such as spatial location, frequency of encounters, larval age or external cues associated with an offspring's location (Bonadonna, Cunningham, Jouventin, Hesters, & Nevitt, 2003; Lank, Bousfield, Cooke, & Rockwell, 1991; Müller & Eggert, 1990; Waldman, 1987). Parents should follow the simplest set of rules to optimize costs and benefits between two kinds of possible errors in offspring recognition: (1) caring for unrelated progeny and (2) rejecting their own offspring as recipients of care (Trivers, 1974). For example, indirect rather than direct recognition is expected to evolve when offspring are not likely to move and are deposited in spatially discrete clusters or inside a parent's territory (Sherman et al., 1997; Waldman, 1987). Sex-specific differences in spatial behaviours (e.g. territoriality versus high mobility) and/or reproductive strategies (e.g. choosing versus advertising sex, parental care versus offspring desertion) might thus favour different offspring discrimination strategies in males and females.

Behavioural differences between males and females are common features across most species and across social/environmental contexts. For example, several studies have demonstrated sex differences in species recognition abilities, probably resulting from the differential costs of mismating and hybridization or sex-specific risks of predation (Saetre, Král, & Bureš, 1997; Svensson, Karlsson, Friberg, & Eroukhmanoff, 2007). Regarding offspring discrimination, sex differences have been shown in the razorbill, *Alca torda*, in which care by each parent takes place at different stages of offspring development (Insley, Paredes, & Jones, 2003). Studies on offspring discrimination have mostly focused on highly social vertebrate species with prolonged and complex parental care (Komdeur & Hatchwell, 1999; Krause & Caspers, 2012), which at the same time are considered to possess high cognitive abilities and learning capacities (Byrne & Whiten, 1988; Kummer, Daston, Gigerenzer, & Silk, 1997; but see also Holekamp, 2007). Little is known about offspring discrimination abilities in less social vertebrates, such as amphibians (but see Poelman & Dicke, 2007; Stynoski, 2009). While general kin discrimination and recognition mechanisms have been demonstrated for several amphibian species (Blaustein & Waldman, 1992; Waldman, 2005), the majority of studies have focused on differential behavioural responses towards kin and nonkin among amphibian larvae. In many animals, including amphibians, spatial and reproductive behaviours differ considerably between the sexes. In species with parental care, differential likelihood and costs of misdirected care might thus drive different offspring discrimination strategies in males and females.

We tested this hypothesis in *Allobates femoralis*, a Neotropical poison frog with sex-specific reproductive strategies and spatial behaviour. Males defend territories of about 150 m^2^ (M. Ringler, Ringler, Magaña-Mendoza, & Hödl, 2011) and announce territory ownership by a prominent advertisement call (Hödl, Amézquita, & Narins, 2004; M. Ringler et al., 2011; M. Ringler, Ursprung, & Hödl, 2009). Females occupy perches which are interspersed between male territories (E. Ringler, Ringler, Jehle, & Hödl, 2012). Both sexes are iteroparous and highly polygamous throughout the prolonged reproductive season (Ursprung, Ringler, Jehle, & Hödl, 2011). Under optimal conditions in captivity females can produce a clutch every 8 days (Weygoldt, 1980). Courtship and mating occur in male territories where terrestrial clutches are laid and fertilized in the leaf litter (Montanarin, Kaefer, & Lima, 2011; E. Ringler et al., 2012; M. Ringler et al., 2009; Roithmair, 1992). Females abandon the clutch and return to their perches immediately after oviposition; males neither remain close to the clutches (i.e. egg guarding) nor provide any further care such as egg moistening or active predator defence. After 3 weeks of larval development the tadpoles are generally transported by the father to nearby water bodies (E. Ringler, Pašukonis, Hödl, & Ringler, 2013; Weygoldt, 1980). However, it has been shown that the mother takes over parental duties when the father disappears (E. Ringler, Pašukonis, Fitch, Huber, Hödl, & Ringler, 2015). As soon as the parent positions itself on the clutch the larvae wiggle onto the parent's back and are subsequently transported to widely dispersed water bodies up to a distance of 200 m (E. Ringler et al., 2013).

Considering the differential reproductive strategies and the unequal frequency of parental care in male and female *A. femoralis*, differences in offspring discrimination strategies between the sexes can be expected. As clutches are deposited in male territories, males can generally assume that all clutches inside their territory are their own offspring, and might therefore use a simple discrimination rule such as ‘all clutches inside my territory are mine’. In contrast, females have their clutches dispersed across multiple male territories, which, in general, will also contain clutches of other females. Thus, if females transfer tadpoles when the male disappears, they should be much more selective than males. Tadpole transport is likely to be costly for the carrying individual in terms of energy investment, predation risk and lost potential mating opportunities. During times of absence other males might also try to take over the territory, resulting in serious fights as soon as the former territory owner returns (E. Ringler, M. Ringler & A. Pašukonis, personal observation). Transport of unrelated offspring would impose these costs on either sex, but without yielding any benefits, and thus should be avoided. Specifically, we asked whether males and females discriminate between their own offspring and unrelated young and whether they use direct or indirect cues when transporting tadpoles. Furthermore, we tested whether parental behaviours are only elicited when an individual is predisposed to perform parental care by the presence of its own clutch.

## Methods

We performed a behavioural experiment under controlled laboratory conditions from August 2014 to March 2015 in the animal care facilities at the University of Vienna. Both wild-caught frogs (*N* = 19) from French Guiana and our own captive-bred individuals (*N* = 29) were used for the experiments (see Table A1). All tested individuals were adult and had successfully produced/sired a clutch previously.

### Ethical Note and Housing

All frogs used in this study are part of the ex situ laboratory population of the animal care facilities at the University of Vienna. Permissions for sampling and export of wild-caught frogs were obtained from the responsible French authorities (DIREN: Arrete n° 82 du 10.08.2012 and Arrete n° du 14.01.2013). All experimental procedures were in strict accordance with current Austrian law, approved by the Ethics Committee of the University of Vienna, and followed the ASAB/ABS guidelines for the treatment of animals in behavioural research and teaching. The experiments were noninvasive as they were based on behavioural observations alone and therefore do not fall under the Austrian Animal Experiments Act (§ 2, Federal Law Gazette No. 114/2012).

All experiments were performed in standard glass terraria of equal size (60 × 40 cm and 40 cm high) with identical equipment and furnishing. The floor was covered with pebbles of expanded clay, the back and side walls were covered with xaxim (plates made of dried tree fern stems) and cork mats, and the front was covered with fabric to prevent visual contact between neighbouring terraria and disturbances during maintenance. All terraria contained half a coconut shell, a small plant and a branch as suitable shelters and calling positions. We provided oak leaves as a substrate for oviposition, and a small glass bowl of 12 cm diameter filled with approximately 35 ml of water for tadpole deposition. An automatic raining, heating and lighting system ensured standardized climatic conditions with similar parameters to the natural conditions in French Guiana in all terraria. Frogs were fed with wingless fruit flies every second day. Apart from the transfer of the mating partners to other terraria after oviposition in trials 2 and 3, no further disturbance happened during the experimental trials.

### Experimental Design

**Figure 1:**
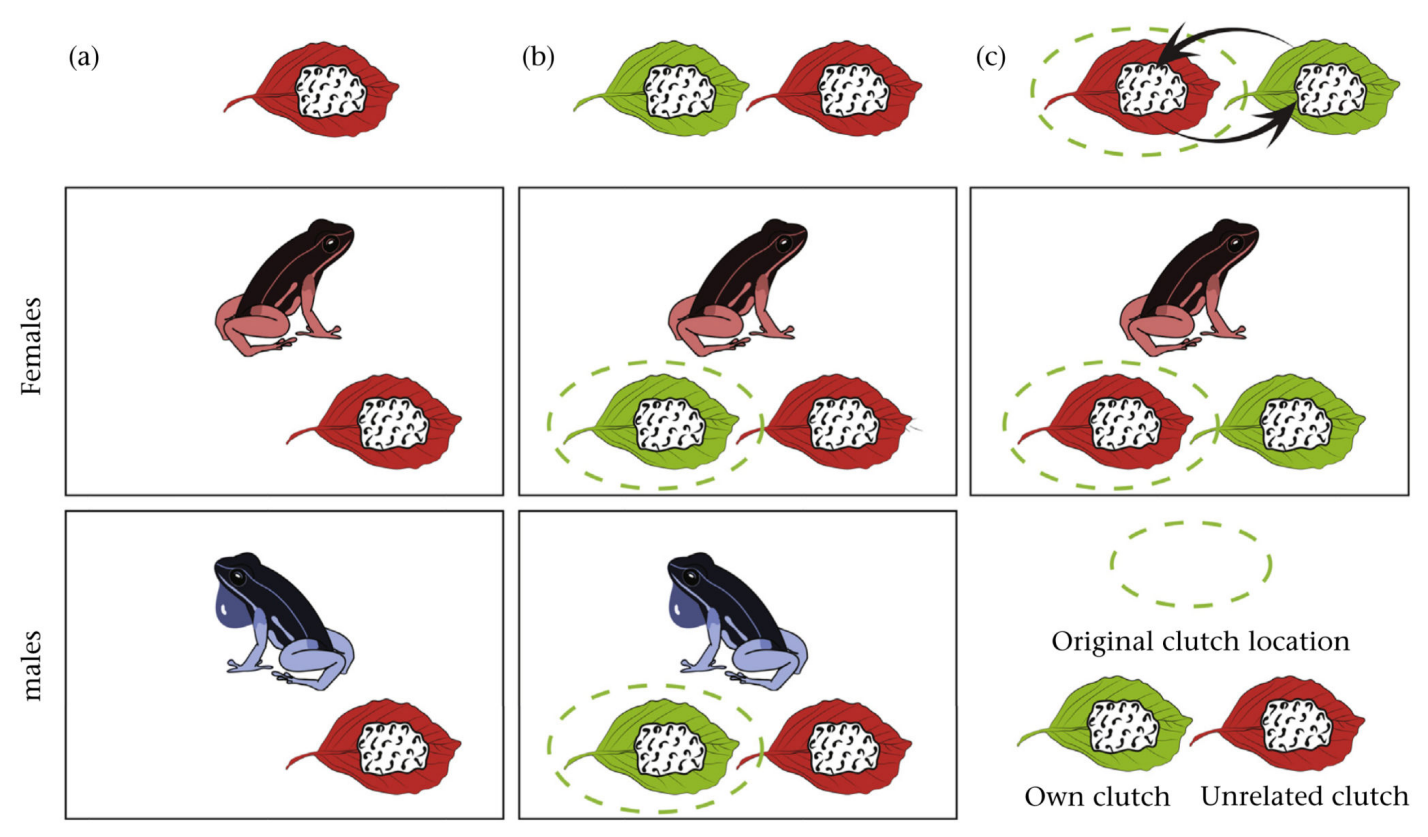
Experimental design. (a) Test 1: unrelated clutches were placed inside the terrarium of males/females that had no own clutch at the same time; (b) test 2: unrelated clutches were added to the terrarium of males/females that already had their own clutches; (c) test 3: unrelated clutches were added to the terrarium of females that already had their own clutches, at the original location of the parent's own clutch, while the latter was moved approximately 20 cm from the original location.

Our experiment, with three test conditions, was designed to identify the use of direct and indirect cues for offspring discrimination, as well as to determine whether the presence of a parent's own clutch is necessary to elicit parental care. In test 1 an unrelated clutch (i.e. a clutch from another pair of frogs) was placed inside the terrarium of an individual that had no clutch of its own at the time. In test 2 an unrelated clutch was added to the terrarium of an individual that already had its own clutch. The unrelated clutch was placed approximately 20 cm away from the parent's own clutch, and the latter's location was not altered. In test 3 we replaced the parent's own clutch with an unrelated clutch and moved the former approximately 20 cm from the original location (see Fig. 1). We matched the parent's own and unrelated clutches by developmental stage (all between Gosner stages 13 and 17; Gosner, 1960) and clutch size. Unrelated clutches did not differ from the parents' own clutches in their number of tadpoles (own: mean ± - SD = 14.2 ± 4.3; unrelated: mean ± SD = 14.7 ± 5.9; paired *t* test: *N* = 30, *t*_29_ = −0.44, *P* = 0.663) in tests 2 and 3.

As it is impossible to manipulate egg clutches directly without substantial destruction, we always moved clutches together with the leaves on which they were deposited. In trial 2 the leaf with the parent's own clutch was also slightly lifted and then placed back at its exact original location, to exclude handling biases. Individuals that participated in test 1 were kept isolated by removing the previous partner for at least 3 weeks prior to testing and until they had transported any remaining clutches. In tests 2 and 3 we permitted pairs to mate and produce one clutch, and a few days after oviposition the respective other partner was removed and the unrelated clutch was added. To keep required sample sizes as small as possible, we started by conducting tests 1 and 2, while test 3 started only after we identified whether males and females discriminate between their own and unrelated clutches. Given that most males transported unrelated clutches in trials of both tests 1 and 2, we only used females in test 3. All individuals were tested only once (*N* = 46), or after a break of at least 3 months before being tested again (*N* = 2). However, the latter two individuals were not used in the same test twice, neither were their data points directly compared in any statistical analysis. We checked terraria daily and recorded which clutches were transported to the water bodies. In cases where parents' own or unrelated clutches failed to develop, suffered from fungus infection or occasional slug predation the trial was stopped and excluded from further analyses. If clutches were not transported within 4 weeks after oviposition and dried up, this was counted as ‘no transport’. The ratios of successful tadpole transport events versus ‘no transport’ were then compared between tests and sexes, respectively, using Fisher's exact test, which is particularly robust and conservative if sample sizes are small. Alpha for rejection of null hypotheses was set a priori at *P* < 0.05.

## Results

**Figure 2:**
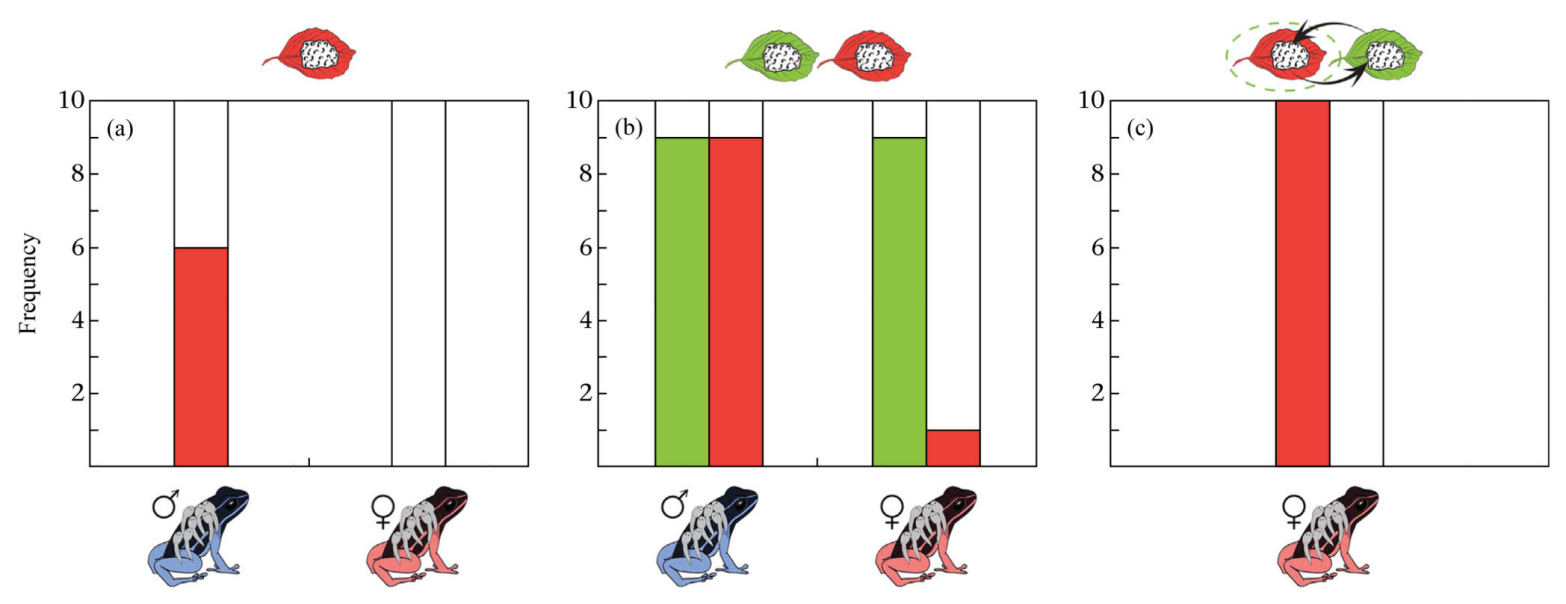
Number of clutches transported by males and females in (a) test 1, (b) test 2 and (c) test 3. Green bars indicate the number of the parents' own clutches and red bars the number of unrelated clutches that were transported by male and female *A. femoralis*. White bars indicate the number of clutches that were not transported.

In test 1, 60% of males (6/10) but no female (0/10; Fig. 2a) transported tadpoles of unrelated clutches (Fisher's exact test: *P* = 0.011). In test 2, 90% of males (9/10; Fig. 2b) transported both their own and unrelated clutches. Females did not transport unrelated clutches, but transported their own clutches in 90% of cases (9/10). Only in a single case (1/10) did a female transport both her own clutch and the unrelated one, while another female (1/10) refused to carry both clutches (Fig. 2b). Thus, in test 2 tadpole transport of unrelated clutches also differed significantly between the sexes (Fisher's exact test: *P* = 0.001). Females in general did not transport tadpoles of unrelated clutches in tests 1 and 2 (no own clutch: 0/10; with own clutch: 1/10). By contrast, in test 3 all females (10/10) transported unrelated clutches when put in the original location of their own clutch, while they did not transport any of their own clutches (0/10) that were moved from their original location (Fig. 2c).

## Discussion

We found different offspring discrimination strategies in male and female *A. femoralis* during parental care. While males also transported unrelated tadpoles, females discriminated between their own offspring and unrelated young according to the exact spatial location of the clutch.

Males in general transported all tadpoles that were placed inside their terraria. They even transported tadpoles in the absence of their own clutch (i.e. if they had not sired a clutch previously), but then they picked up unrelated tadpoles less often than males that had their own clutch present. However, the fact that even 60% of those males without their own clutch transported unrelated tadpoles suggests a strong motivation in *A. femoralis* males to transport all encountered larvae to water, at least all conspecific ones situated inside a male's territory.

In a recent study on tadpole transport behaviour in this species (E. Ringler et al., 2013) males were observed to occasionally transport clutches of neighbouring males (four of 119). These cases were probably caused by shifts in territory boundaries and show that, although rare, transport of unrelated tadpoles also occurs under natural conditions, at least in this study population which had a density of about 23 males/ha (M. Ringler, Hödl, & Ringler, 2015). These field observations are in line with the results of the present study. We suggest that strong male territoriality favoured the observed behavioural pattern in males. Males actively defend their territories against male intruders (Narins, Grabul, Soma, Gaucher, & Hödl, 2005; M. Ringler et al., 2011) and clutches are exclusively laid within territory boundaries (Montanarin et al., 2011; Roithmair, 1992). Thus even when clutches might have changed their position slightly due to naturally occurring disturbances, such as other animals trespassing, males can still generally expect that all clutches inside their territory are their own. Consequently, there is no need for males to discriminate between the clutches inside their territory. In turn, the costs of accidentally rejecting their own clutch would presumably be higher than occasionally transporting unrelated tadpoles (cf. brooding birds that accept parasitic eggs, Rothstein, 1975). However, the simple discrimination rule ‘all clutches inside my territory are mine’ is only feasible when territories are stable and shifts in boundaries are rare. We cannot exclude that males actually recognize their own offspring but still decide to transport all tadpoles they encounter. However, given the expected costs associated with tadpole transport in terms of energy expenditure, predation risk, potential territory take-overs and lost mating opportunities, we consider this scenario unlikely. Follow-up experiments in the field are needed to corroborate that males differentiate between clutches that are ‘inside’ or ‘outside’ their territory boundaries. Furthermore, future studies should investigate tadpole transport behaviour in natural populations with high male densities, where territory shifts are probably more common.

Females only transported tadpoles when they had recently produced a clutch and accepted clutches based on their exact location. Even their own clutches that were relocated by only 20 cm were not transported. As tadpoles cannot move between clutches, the exact spatial location of the clutch is probably sufficient to allow mothers to approach their own offspring (cf. Sherman et al., 1997; Waldman, 1987). The high polygyny and clutch deposition in the males' territories can confront females with clutches of several other females in close proximity to their own. The likelihood of accidentally picking up the wrong clutch is therefore much higher in females than in males. Consequently, establishing rules for offspring discrimination is more complex for females. Moreover, misdirected care can be expected to have differential costs to the sexes. As males generally transport any clutch inside their territory, transporting an unrelated clutch only leads to additional costs for the carrying male, but will hardly impact his remaining offspring. In contrast, females only transport tadpoles as a specific behavioural response triggered by their partner's disappearance. Consequently, the accidental transport of an unrelated clutch would automatically result in their own clutch not being transported, as from the female's perspective her compensatory duties have already been fulfilled. Thus, the costs of misdirected care are much higher for female than male *A. femoralis.*

Peterson (2000) suggested that if the probability of nest confusion is high, direct egg recognition should be favoured. In our study, female *A. femoralis* did not discriminate between clutches based on specific clutch-related cues, but were spatially accurate when transporting clutches. We speculate that direct clutch recognition is not feasible in *A. femoralis* because of insufficient phenotypic variation between clutches (cf. Tibbets & Dale, 2007) and the ontogenetic change from zygotes to the hatched tadpoles. Although scent-based offspring discrimination mechanisms are known from many vertebrate species (Johnston, Muller-Schwarze, & Sorensen, 1999; Yamazaki, Beauchamp, Curran, Bard, & Boyse, 2000), apparently odour cues of tadpoles are not used for offspring discrimination in *A. femoralis* (see also Schulte & Veith, 2014). In recent studies, remarkable orientation and spatial learning abilities have been demonstrated for two dendrobatid species. In a visual discrimination task *Dendrobates auratus* males used visual cues for spatial orientation and were also able to update their visual associations in a reversal learning task (Liu, Day, Summers, & Burmeister, 2016). In *A. femoralis* males, the high accuracy and precision during homing behaviour after experimental translocation and the loss of this orientation ability in unfamiliar habitat indicate the relevance of spatial learning for flexible navigation in their local area (Pašukonis et al., 2013; Pašukonis, Warrington, Ringler, & Hödl, 2014). Males repeatedly commute between home territories and tadpole rearing sites and presumably possess very detailed knowledge of the surrounding area. Our study suggests that females also have very precise spatial knowledge and remember the locations of specific clutches over the course of weeks. This is particularly surprising as females will probably have produced one or two further clutches with other males during the development of the initial clutch (E. Ringler et al., 2012) and as female tadpole transport is rare in the field (7%, E. Ringler et al., 2013). In this context, future studies should test whether the simultaneous presence of multiple clutches reduces a female's relocation ability. Further studies are also needed to identify whether females learn and remember certain features in their complex and changing habitat and how they manage to relocate their clutches after several weeks.

Previous studies in other dendrobatid species have shown that *Ranitomeya amazonica* (previously *Dendrobates ventrimaculata* in this location; Poelman & Dicke, 2007) males do not discriminate between offspring and unrelated young, whereas *Oophaga pumilio* females do (Stynoski, 2009). Mothers of *O. pumilio* provisioned tadpoles regardless of tadpole identity, but were highly sensitive to location as they did not provision tadpoles that were moved 2 cm to an adjacent cup. Stynoski (2009) hypothesized that the reason why *O. pumilio*, but not *R. amazonica*, discriminate between offspring and unrelated young, is that *O. pumilio* invest more in their offspring, both transporting and feeding tadpoles, than *R. amazonica* which only transport tadpoles. Our findings do not support the idea that in *A. femoralis* offspring discrimination has evolved in association with high levels of parental investment. Offspring discrimination seems to be linked to the risk of choosing the ‘wrong’ clutch (e.g. when clutches of different individuals are found in close spatial proximity), supporting the hypothesis that offspring discrimination strategies are shaped by the likelihood and costs of misdirected care (cf. Beecher, 1991). In fact, this hypothesis is also corroborated by the findings of Stynoski (2009) and Poelman and Dicke (2007), as female *O. pumilio* home ranges overlap, whereas male *R. amazonica* defend well-defined reproductive territories.

In some species, offspring recognition is not used until some minimum level of parental investment has occurred (Lefevre, Montgomerie, & Gaston, 1998; Mateo, 2006; Müller & Eggert, 1990). In the present study males even transported tadpoles when they had no clutches of their own, while females did so only when they had recently produced a clutch. Parental behaviour is presumably strongly influenced by the hormonal status of an individual (Hunt, Hahn, & Wingfield, 1999; Kindler, Bahr, Gross, & Philipp, 1991; Neumann, 2008). Our findings suggest that females are predisposed to perform parental care only after oviposition and probably also use temporal information for assessing parentage of a given clutch. In turn, the continuous calling of *A. femoralis* males during the rainy season might induce and maintain a certain hormonal state in males, where they remain in a ‘reproductive mode’ which includes mating but also tadpole transport. However, the slightly lower tadpole transport rate in males that had not recently sired a clutch indicates hormonal and/or motivational changes are also induced by siring a clutch in males. Further studies, using larger sample sizes and possibly also hormonal analyses, are needed to more accurately investigate motivational changes according to tadpole transport induced by courtship and mating in males and females.

### Conclusions

We have shown that different offspring discrimination strategies have evolved in male and female *A. femoralis*, probably as a response to different risks of misdirected care between the sexes. The high and low selectivity in males and females, respectively, regarding tadpole transport reflect differences in the most reliable and efficient solutions for differentiating between their own and unrelated tadpoles in the two sexes. Future studies should investigate how different uncertainties, such as stability of male territories or higher levels of polygyny, influence discrimination strategies in males and females.

## Supplementary Material

Appendix
