# Supplementary material for: Sex-specific offspring discrimination reflects respective risks and costs of misdirected care in a poison frog
Source: Anim Behav. Author manuscript; Available in PMC 2017 Feb 22. (PMC5321237; doi:10.1016/j.anbehav.2016.02.008)
Supplement: Appendix [file NIHMS69668-supplement-Appendix.pdf]

APPENDIX

Table A1
Detailed list of experimental trials

Trial	Date	Test	Individual	Sex	Origin	Own clutch	No. of tp own	No. of tp unrelated	TT own	TT unrelated
1	29 Jul 2014	2	femo07	M	W	1	10	8	1	1
2	29 Jul 2014	2	femo25	M	W	1	9	9	1	1
3	29 Jul 2014	2	femo40	M	C	1	13	16	1	1
4	31 Jul 2014	1	femo05	M	W	0	N/A	15	N/A	1
5	31 Jul 2014	2	femo03	M	W	1	10	9	1	1
6	01 Aug 2014	1	femo13	M	W	0	N/A	12	N/A	1
7	04 Aug 2014	1	femo10	F	W	0	N/A	16	N/A	0
8	05 Aug 2014	1	femo28	F	W	0	N/A	9	N/A	0
9	06 Aug 2014	1	femo04	F	W	0	N/A	14	N/A	0
10	13 Aug 2014	1	femo22	M	W	0	N/A	16	N/A	1
11	13 Aug 2014	1	femo23	M	W	0	N/A	12	N/A	1
12	28 Aug 2014	2	femo06	F	W	1	14	13	1	0
13	28 Aug 2014	2	femo24	M	W	1	18	13	1	1
14	28 Aug 2014	2	femo43	M	C	1	12	15	1	1
15	03 Sept 2014	2	femo47	F	C	1	9	24	1	0
16	05 Sept 2014	1	femo51	M	C	0	N/A	12	N/A	1
17	08 Sept 2014	2	femo16*	F	W	1	17	9	1	0
18	01 Oct 2014	2	femo32	F	W	1	12	11	0	0
19	07 Oct 2014	2	femo29*	F	W	1	20	15	1	0
20	07 Oct 2014	2	femo30	F	W	1	14	15	1	0
21	07 Oct 2014	2	femo39	F	C	1	12	20	1	0
22	20 Oct 2014	2	femo55	F	C	1	14	22	1	0
23	30 Oct 2014	1	femo78	F	C	0	N/A	14	N/A	0
24	30 Oct 2014	1	femo81	F	C	0	N/A	10	N/A	0
25	30 Oct 2014	1	femo86	F	C	0	N/A	23	N/A	0
26	11 Nov 2014	2	femo66	F	C	1	10	8	1	0
27	11 Nov 2014	2	femo35	M	W	1	17	13	1	1
28	11 Nov 2014	2	femo45	M	C	1	11	15	0	0
29	25 Nov 2014	1	femo68	F	C	0	N/A	11	N/A	0
30	10 Dec 2014	1	femo12	F	W	0	N/A	13	N/A	0
31	10 Dec 2014	1	femo42	M	C	0	N/A	21	N/A	0
32	10 Dec 2014	1	femo44	M	C	0	N/A	21	N/A	0
33	10 Dec 2014	1	femo59	M	C	0	N/A	19	N/A	1
34	15 Dec 2014	1	femo74	M	C	0	N/A	10	N/A	0
35	19 Dec 2014	2	femo49	F	C	1	19	11	1	1
36	19 Dec 2014	2	femo48	M	C	1	13	13	1	1
37	30 Dec 2014	1	femo02	M	W	0	N/A	17	N/A	0
38	12 Jan 2015	3	femo16*	F	W	1	10	15	0	1
39	12 Jan 2015	3	femo29*	F	W	1	6	14	0	1
40	12 Jan 2015	3	femo57	F	C	1	15	12	0	1
41	12 Jan 2015	3	femo63	F	C	1	14	11	0	1
42	12 Jan 2015	3	femo73	F	C	1	21	14	0	1
43	15 Jan 2015	1	femo67	F	C	0	N/A	10	N/A	0
44	29 Jan 2015	1	femo76	F	C	0	N/A	14	N/A	0
45	29 Jan 2015	3	femo75	F	C	1	17	29	0	1
46	02 Feb 2015	3	femo53	F	C	1	25	18	0	1
47	02 Feb 2015	3	femo69	F	C	1	20	33	0	1
48	05 Feb 2015	3	femo80	F	C	1	18	15	0	1
49	23 Feb 2015	2	femo84	M	C	1	13	11	1	1
50	17 Mar 2015	3	femo83	F	C	1	13	10	0	1

Trial: consecutive trial number; date: date when experiment was started; individual: ID of the tested individual, asterisks indicating multiply tested individuals; sex: M = male, F = female; origin: w = wild-caught individual, c = captive-bred individual; own clutch: presence of the parent's own clutch, 0 = no, 1 = yes; no. of tp own: number of tadpoles in the parent's own clutch; no. of tp unrelated: number of tadpoles in the unrelated clutch; TT own: tadpole transport of the parent's own clutch, 0 = no, 1 = yes; TT unrelated: tadpole transport of the unrelated clutch, 0 = no, 1 = yes.
